# Mouse Studies to Shape Clinical Trials for Mitochondrial Diseases: High Fat Diet in Harlequin Mice

**DOI:** 10.1371/journal.pone.0028823

**Published:** 2011-12-13

**Authors:** Manuel Schiff, Paule Bénit, Riyad El-Khoury, Dimitri Schlemmer, Jean-François Benoist, Pierre Rustin

**Affiliations:** 1 INSERM, U676, Paris, France; 2 Université Paris 7, Faculté de Médecine Denis Diderot, IFR02, Paris, France; 3 APHP, Hôpital Robert Debré, Centre de Référence Maladies Héréditaires du Métabolisme, Paris, France; 4 APHP, Hôpital Robert Debré, Laboratoire de Biochimie, Paris, France; University of Bremen, Germany

## Abstract

**Background:**

Therapeutic options in human mitochondrial oxidative phosphorylation (OXPHOS) diseases have been poorly evaluated mostly because of the scarcity of cohorts and the inter-individual variability of disease progression. Thus, while a high fat diet (HFD) is often recommended, data regarding efficacy are limited. Our objectives were 1) to determine our ability to evaluate therapeutic options in the Harlequin OXPHOS complex I (CI)-deficient mice, in the context of a mitochondrial disease with human hallmarks and 2) to assess the effects of a HFD.

**Methods and Findings:**

Before launching long and expensive animal studies, we showed that palmitate afforded long-term death-protection in 3 CI-mutant human fibroblasts cell lines. We next demonstrated that using the Harlequin mouse, it was possible to draw solid conclusions on the efficacy of a 5-month-HFD on neurodegenerative symptoms. Moreover, we could identify a group of highly responsive animals, echoing the high variability of the disease progression in Harlequin mice.

**Conclusions:**

These results suggest that a reduced number of patients with identical genetic disease should be sufficient to reach firm conclusions as far as the potential existence of responders and non responders is recognized. They also positively prefigure HFD-trials in OXPHOS-deficient patients.

## Introduction

Mitochondria play a vital role by producing most of the cellular ATP via oxidative phosphorylation (OXPHOS) [1]. OXPHOS is responsible for transferring energy from macronutrients to ATP through a sequence of coordinated reactions by which macronutrients are oxidized, oxygen is reduced to water, and ADP is phosphorylated to ATP. OXPHOS is catalyzed by the respiratory chain (RC) which comprises the proton-pumping respirasome associating complexes I, III, and IV, and a number of dehydrogenases including complex II and the ATP synthasome [2].

Inherited mitochondrial dysfunction causes heterogeneous and often multisystemic disorders (mt or OXPHOS diseases) that preferentially affect tissues with high-energy demands such as brain [3], muscle and heart, although any organ can be affected at any age [4], [5]. Among them, defects of the RC complex I (CI) are the most frequent [6].

While major progress has been made in the identification of the molecular bases at the origin of OXPHOS defects including CI deficiencies [7], no treatment is currently available [8]. Supportive care includes nutritional modulations (specifically fat-enriched diet) [9]. However, this scheme is mostly based on theoretical assumptions or circumstantial reports. Experimental data that would support it are still lacking. Harlequin (Hq) mouse is a faithful model for CI deficiency, mimicking the human disease by the variability of the severity and the progression rate of symptoms [10]. This was a strong incentive to evaluate diet effect in Hq mice under realistic conditions for a mt disease to prefigure human trials.

The Hq phenotype is due to a proviral insertion in the X-linked gene encoding the mt protein Apoptosis Inducing Factor (AIF) [11] causing an 80% decrease in the AIF protein. Hemizygous males and homozygous females exhibit a phenotype of variable (albeit comparable) severity [10] with growth retardation, progressive cerebellar ataxia, early fur abnormalities, optic tract dysfunction with pigmentary retinopathy, and increased risk of heart failure [11], [12]. We previously established that Hq mice are partially CI-defective [13] with a tissue specific and variable progressive pattern [10]. Accordingly, CI deficiency was shown to be the cause of the Hq phenotype [13], [14]. As it reproduces many features of human mt diseases, and because of the progressiveness of its phenotype with a symptom-free interval, the Hq model is an appropriate tool for investigating treatments aimed at fighting OXPHOS diseases [15]. We have shown that weight loss, baldness and cerebellar ataxia were correlated parameters whose time course closely reflected disease progression [10]. Notably, the first *AIF* mutations were reported in 2 related (first cousins) male patients affected with a typical and severe mt encephalomyopathy [16]. Very recently, two brothers with prenatal ventriculomegaly and severe mt encephalomyopathy were reported with a mutation in *AIF* [17]. These reports of human *AIF* mutations validate *AIF* depletion as a cause of human mt OXPHOS disease.

Fatty acids (FA) are well-known ligands of the peroxisome proliferator-activated receptors (PPARs) [18], [19], [20] which participate in the control of mt biogenesis in a number of tissues [21], [22], [23], [24]. The theoretical rational to use FA in mt diseases is therefore well established as well as their mechanisms of action on mitochondria.

Taking advantage of an available faithful model for mt disease and of a promising therapeutic option (strong rational and circumstantial reports [25], [26], [27], [28]), we decided to test high fat diet (HFD) in a placebo-controlled trial on a population of mice. However, before launching a long and expensive animal study, we verified that FA had indeed a positive impact in CI- deficient human fibroblasts.

## Results

### Palmitate protects human Complex I deficient fibroblasts from death

**Figure 1:**
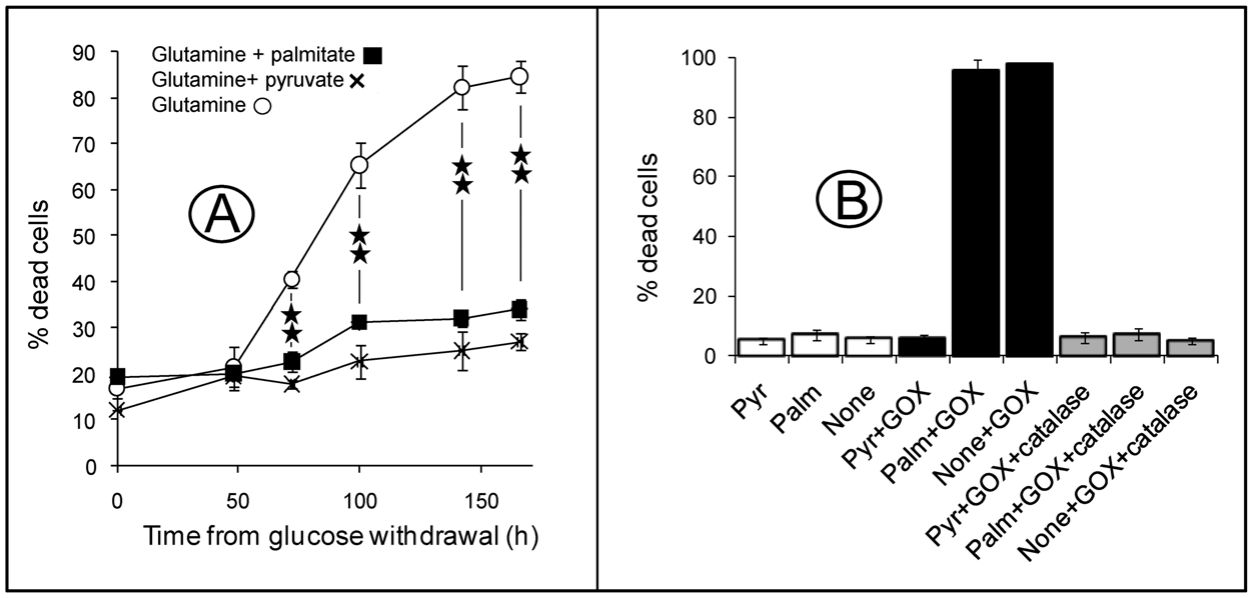
Protection from cell death triggered by either glucose withdrawal in patient 1 (P1) cells (A) or hydrogen peroxide addition in control cells (B) afforded by palmitate or pyruvate. A: In Patient 1 (P1) cells, death occurred within 72 hours after glucose withdrawal, when cells were cultured in the presence of glutamine (open circles). When cells were cultured in the presence of 100 µM palmitate (dark squares) or 2 mM pyruvate (crosses), these were significantly protected from death. B: Control cells were counted 24 hours after the addition of 2 mM pyruvate (Pyr) or 100 µM palmitate (Palm) in standard DMEM 4.5 g/L glucose, 4 mM glutamine medium (none) with or without 0.001 mg/mL glucose oxidase or glucose oxidase and 350 ng/mL catalase. Pyruvate but not palmitate protects against H_2_O_2_-induced cell death. Values are means ± SEM from triplicate experiments. **: p<0.01 (Student's two-tailed *t* test). Experimental procedures as described under Materials and methods.

A number of crucial features in cells including ATP synthesis, calcium handling and ROS generation presumably depend on mt CI function. Thus it was not surprising that under our experimental growth conditions, most cells with severe CI deficiency died upon glucose withdrawal from the culture medium. Notably, supplementing the growth medium with the saturated fatty acid palmitate (100 µM palmitate, 1 mM L-carnitine, 0.5 mg/mL BSA) protected a number of CI-deficient cells from death triggered by glucose withdrawal. This effect was particularly spectacular in one *NDUFS1* (del222/D252G) [29] mutant cell line (patient 1) for which death occurred within 72 hours after glucose withdrawal with full protection afforded by 100 µM palmitate (Fig. 1A). These cells were still viable after 8 days of culture in the absence of glucose but in the presence of palmitate. In two other CI-defective cell lines (harbouring mutations in *NDUFS1* [del<44, 2cM/M707V], patient 2 [29] and *NDUFS4* [IVS1nt –1, G→A], patient 3 [30]), death occurred within 170 hours after glucose withdrawal again with full protection by palmitate (Table 1). FA death-preventive effect was not observed in cells cultured with L-carnitine alone or BSA alone (Table 2) confirming that the protective effect was exclusively attributable to palmitate itself. A similar protection was obtained with pyruvate (Fig. 1A) but not with succinate (Table 2). There was no significant effect on cell survival/proliferation of glucose withdrawal, palmitate, succinate or pyruvate supplementation in control cell lines (Tables 2 and 3). Protection from death afforded by pyruvate in culture medium of RC deficient cells may partly be due to the well-known peroxide-scavenging activity of this ketoacid [31]. Accordingly, pyruvate fully protected control cells from death resulting from the H_2_O_2_ produced by glucose oxidase added to the culture medium (Fig. 1B). No such protection from H_2_O_2_ insult could be observed when adding palmitate to the culture medium. Under all conditions, a full protection was provided by adding catalase (Fig. 1B).

**Table 1:** Palmitate-induced death protection in two other CI-deficient human fibroblasts.

		% dead cells	
Patients	Culture medium	0 h	170 h
**P2 (** ***NDUFS1*** **)**	Glutamine	15±2	75±3.5
	Glutamine+palmitate	20±3.2	25±2.7
**P3 (** ***NDUFS4*** **)**	Glutamine	20±1.2	70±4
	Glutamine+palmitate	17±2	18±3

Values are means ± SEM from triplicate experiments; h: time from glucose withdrawal in hours.

**Table 2:** Effect of bovine serum albumin (BSA), L-carnitine, (BSA+L-carnitine+palmitate) and succinate on glucose withdrawal-induced cell death in patient 1 (P1) and control (Ctrl) cells.

	% dead cells (P1/Ctrl)		
Culture medium	0 h	72 h	150 h
Glutamine	15±3/9±1	40±1.7/12±2	85±3/15±2
Glutamine+BSA	15±3/8±1	45±2/12±2	90±5/16±3
Glutamine+L-C	10±2/10±1	50±4/12±3	88±4/18±2
Glutamine+BSA+L-C+palm	19±3/12±1	22±2/12±2	22±3/20±2
Glutamine+succinate	15±3/10±1	45±2/12±2	89±5/19±1

Values are means ± SEM from triplicate experiments; h: time from glucose withdrawal in hours; L-C: L-carnitine; palm: palmitate.

**Table 3:** Absence of cell death in control (Ctrl) cells after glucose withdrawal, palmitate or pyruvate supplementation.

	% dead cells				
Culture medium	0 h	48 h	100 h	142 h	166 h
Glutamine	10±0.5	10.9±1.5	15±1.5	17.3±2	18±2.6
Glutamine+palmitate	11.8±0.7	13.7±1.2	15.3±1.9	14.6±3.4	16.5±0.5
Glutamine+pyruvate	12.8±0.8	15.3±3.8	13.4±3.4	16±1.7	17.8±0.6

Values are means ± SEM from triplicate experiments; h: time from glucose withdrawal in hours.

**Figure 2:**
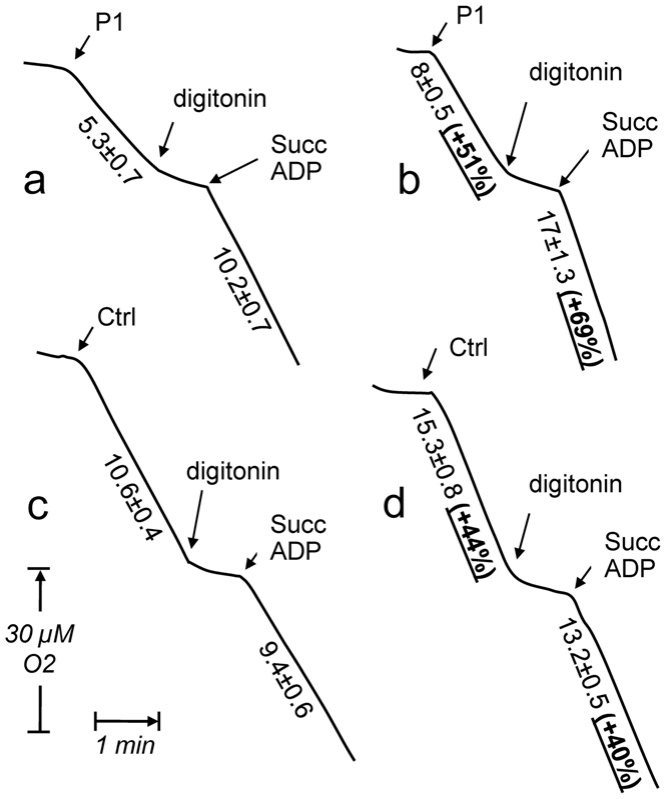
Respiration and mitochondrial substrate oxidations in patient 1 (P1) and control cells grown in the absence or presence of palmitate. Intact P1 cell respiration was 51% higher in cells grown in the presence of palmitate (comparison traces a and b). A subsequent addition of 0.004% digitonin made cells (approximately 2.10^6^ cells) permeable to small molecules including respiratory substrates, with the subsequent dilution of respiratory substrates and progressive arrest of oxygen consumption. Adding 10 mM succinate (Succ) and 0.4 mM ADP triggered a rapid oxygen uptake which was 70% higher for P1 cells grown with palmitate (b). A similar effect, albeit less pronounced of growing cells in the presence of palmitate could be observed using control cells (comparison traces c and d). Numbers along the traces are nmol/min/mg protein. Values are means ± SEM from triplicate experiments. Experimental procedures as described under Materials and methods.

**Figure 3:**
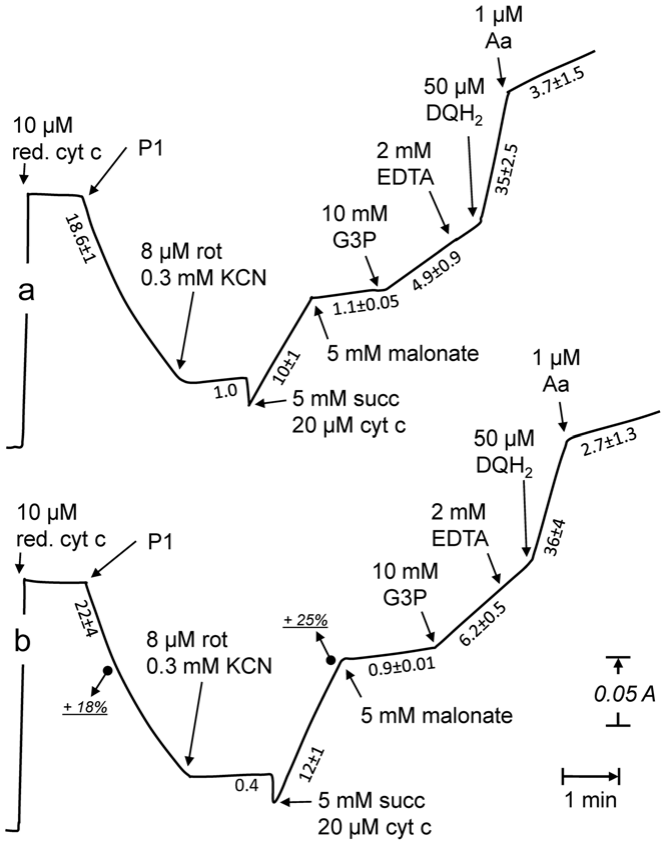
Respiratory chain activities in patient 1 (P1) cells grown in the absence or presence of palmitate. Cytochrome oxidase, estimated by the oxidation rate of reduced cytochrome *c* (downward deflexion), was about 20% increased in P1 cells cultured with palmitate (comparison traces a and b). Adding cyanide (KCN) fully inhibited the oxidation of cytochrome *c*. The subsequent addition of succinate (succ), in the presence of rotenone (rot) and additional oxidized cytochrome *c*, triggered a cytochrome *c* reduction (upward deflexion) which was essentially sensitive to malonate. This succinate-cytochrome *c* reductase activity, measuring complex II+III activity, was about 25% higher in P1 cells grown in the presence of palmitate (b). Adding glycerol 3-phosphate (G3P) triggered a new increase of cytochrome *c* reduction, corresponding to the activity of the glycerol 3-phosphate cytochrome *c* reductase (glycerol 3-phosphate dehydrogenase+complex III), 40% higher in cells grown with palmitate (b). The subsequent addition of decylubiquinol (DQH_2_) in the presence of EDTA triggered a rapid cytochrome *c* reduction which was 90% sensitive to antimycin (Aa). This antimycin-sensitive quinol-cytochrome *c* reduction (complex III activity) did not differ between cells grown in the absence or presence of palmitate. Numbers along the traces are nmol/min/mg protein. Values are means ± SEM from triplicate experiments. Experimental procedures as described under Materials and methods.

In the *NDUFS1* mutant fibroblasts that significantly responded to palmitate (patient 1), the protection from death was associated with increase in RC activities demonstrated by polarographic and spectrophotometric assays (Fig. 2 and 3). Noticeably, the palmitate-induced death protection of these CI-deficient fibroblasts could not be ascribed to an uncoupling effect, as the very low respiration was basically not controlled by the phosphorylation process in the absence of palmitate (respiratory control: 1.1±0.1). If anything, palmitate in culture medium improved respiratory control (1.3±0.1) possibly by increasing respiration rate.

The clear effect of palmitate on cultured CI-deficient cells was an incentive to extend the investigations on the potential effect of long-chain saturated fatty acids using the CI-deficient Harlequin mouse. With this aim, we studied the effect of a nine fold saturated fatty acids-enriched food (high fat diet; HFD) on the course of the Hq disease focusing on parameters that we have previously found to be relevant for Hq disease progression.

### HFD-fed Hq animals exhibit a slower progression of the neurodegenerative phenotype

**Figure 4:**
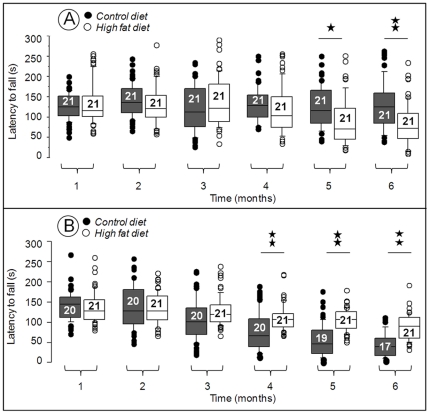
Box-and-whisker plot of rotarod scores as a function of diet (HFD versus CD) according to age in Harlequin (Hq) and wild type (WT) animals. The results of three tests per animals performed monthly from one to six months of age are shown with the number (n) of animals indicated in each box. The vertical bars represent the range of values from the 10^th^ to the 90^th^ percentile; the boxes represent the values from the 25^th^ to the 75^th^ percentile; the horizontal lines represent the median value for each group of animals. Isolated circles represent all observations (in triplicates) below the 10^th^ percentile or above the 90^th^ percentile. *: p<0.05; **: p<0.01 (Student's two-tailed *t* test). HFD-fed WT mice exhibit significantly worse performances than the WT mice fed the CD, from 5 months of age due to their obese phenotype (A). From 4 months of age, HFD-fed Hq mice do significantly better than the Hq fed the CD with a significantly lower progression of the rotarod scores loss (B).

**Figure 5:**
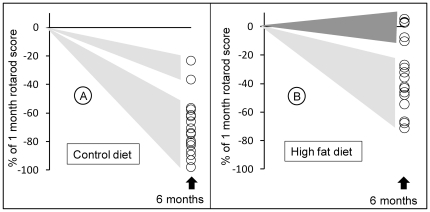
Percentage of individual loss or gain on the rotarod at 6 months of age relative to 1 month of age in Harlequin mice on control (A) or high fat diet (B). Note the presence of a subset of 6 HFD-Harlequin-responder individuals (dark grey).

At one month of age, wild type (WT) animals fed with the control diet (CD) obtained on average a score of 126±23 in the rotarod test. This score remained essentially unchanged up to six months of age (Fig. 4A; closed symbols). There was a tendency towards lower scores in HFD-fed WT animals from 4 months of age which became more obvious at 5 months (Fig. 4A; open symbols), presumably due to HFD-induced obesity [32]. In comparison, Hq animals fed with the CD who had a score of 139±23 comparable to WT animals at 1 month of age, lost about 17 points/month between 1 and 6 months: −62 points between 1 and 4 months and −40 points between 4 and 6 months (Fig. 4B; closed symbols). The rotarod scores were significantly improved by the high fat diet. At 1 month of age, HFD-fed Hq animals had an average score of 135±26 similar to WT animals (Fig. 4; comparison A and B) only losing 9 points/month on average: −24 points between 1 and 4 months and −21 points between 4 and 6 months (Fig. 4B; open symbols). By the age of 4 months, rotarod scores of the HFD-fed Hq animals were significantly higher than the scores of CD-fed Hq animals and these better scores were maintained until 6 months of age (Fig. 4B). When analyzing rotarod scores individually, the relative loss in rotarod scores between 1 and 6 months of age ranged from −98% to −23% of the initial value of the CD-fed Hq animals (Fig. 5A) and from −71% to +5% of the initial value of the HFD-fed Hq mice (Fig. 5B). Notably, 2/17 (11%) animals of the CD-fed Hq mice population (Fig. 5A) displayed a slower progression of the disease reflecting the variability of the disease progression previously reported in the Hq mice. Interestingly, 6/21 (29%) of the HFD-fed Hq mice showed a preserved (19% of the individuals) or slightly reduced (≤10% of the initial value, 10% of the individuals) rotarod performance (Fig. 5B, dark grey triangle). This population (29%) of mice protected from disease when measured by rotarod, clearly indicates the presence of a subset of Hq individuals who indubitably respond to HFD.

**Figure 6:**
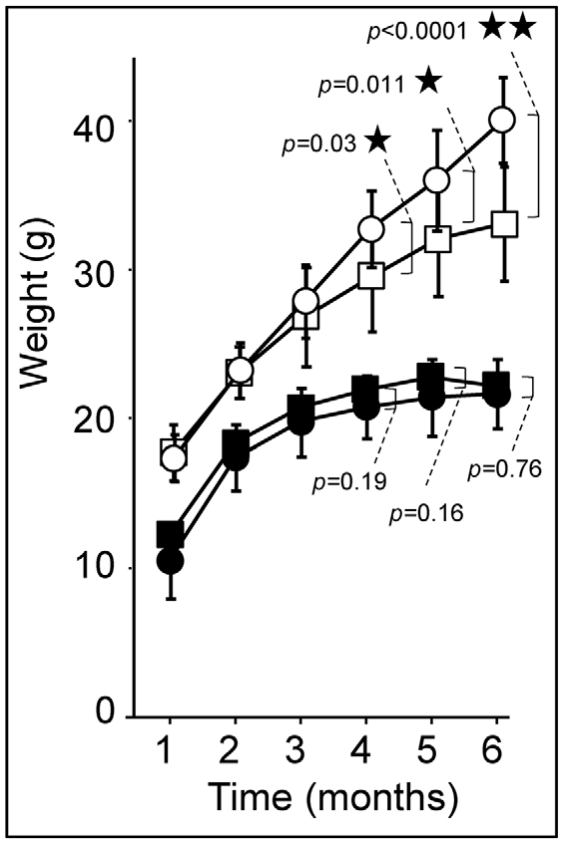
Harlequin mice are spared from the weight gain associated with chronic high fat feeding. Open symbols: wild type animals; dark symbols: Harlequin mice; squares: CD; circles: HFD. *: p<0.05; **: p<0.01 (Student's two-tailed *t* test). Harlequin mice fed the CD (n = 17 to 20); Harlequin mice fed the HFD (n = 21); WT mice fed the CD (n = 21); WT mice fed the HFD (n = 21).

**Figure 7:**
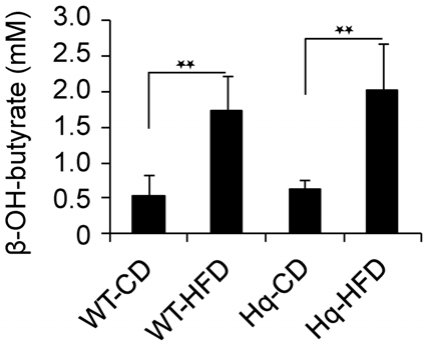
HFD-fed individuals exhibit a higher blood level of β-hydroxybutyrate than the CD-fed individuals. Blood level of β-hydroxybutyrate (β-OH-butyrate) was determined at 6 months of age, after 24 hours of fasting in 5 CD-fed WT mice, 5 HFD-fed WT mice, 10 CD-fed Hq mice and 10 HFD-fed Hq mice. **: p<0.01 (Student's two-tailed *t* test).

**Figure 8:**
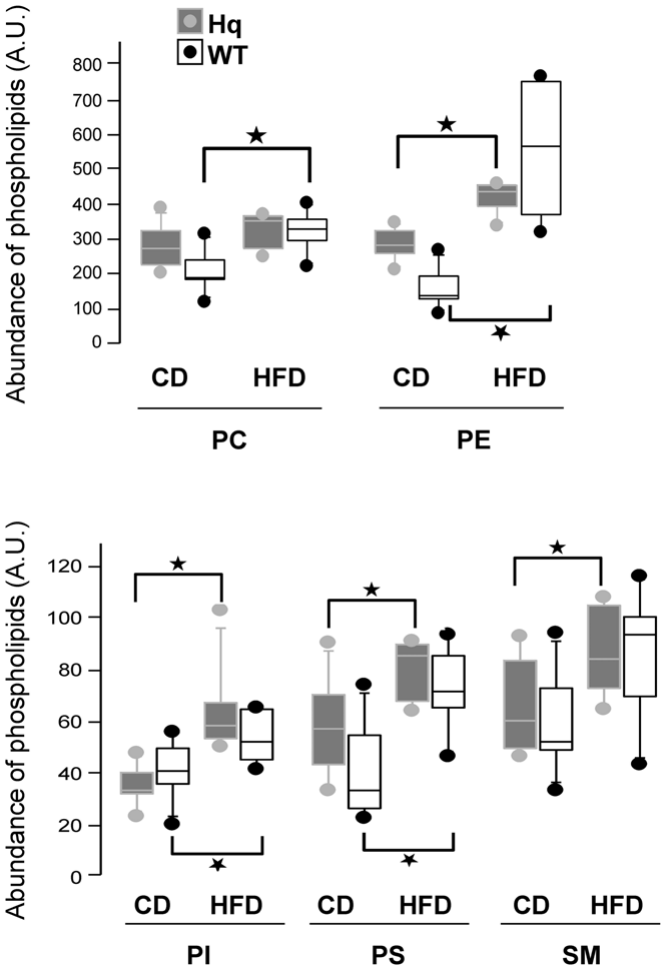
Box-and-whisker plot of different classes of phospholipids in cerebellum membranes. The abundance of various phospholipids is expressed in arbitrary units (A.U.) related to their internal standard and to the cerebellum dry weight (PC, phosphatidylcholine; PE, phosphatidylethanolamine; PI, phosphatidylinositol; PS, phosphatidylserine; SM, sphingomyeline) in 7 WT and 7 Hq individuals. When compared to CD, HFD-fed individuals exhibit significantly higher levels of all the phospholipids in cerebellum except from PC in Hq and SM in WT which are not different between the 2 groups (CD and HFD). The vertical bars represent the range of values from the 10^th^ to the 90^th^ percentile; the boxes represent the values from the 25^th^ to the 75^th^ percentile; the horizontal lines represent the median value. Isolated circles represent all observations below the 10^th^ percentile or above the 90^th^ percentile. *: p<0.05.

It has been previously shown [10] that rotarod scores of the Hq mice were correlated to the animal weights *i.e.* the most severely ataxic individuals (as approximated by the rotarod score) had the lowest weight. We first verified that there was no weight difference between the 2 groups of Hq animals and confirmed that the HFD-fed Hq animals were resistant to HFD-induced obesity observed in the WT animals (Fig. 6) [33]. We also confirmed that Hq animals and the WT mice consumed similar amounts of food per day (at the age of 3 months, the average daily food intake was 4±0.5 g/d or 13.5±1.5 kcal/d either CD or HFD), indicating normal food intake. In keeping with this, we indirectly confirmed correct absorption of the HFD since 1) at 6 months of age, after 24 hours of fasting, the blood level of β-hydroxybutyrate (the major ketone body) was significantly higher in HFD-fed individuals (Hq and WT) than in CD-fed animals attesting for increased fatty acid β-oxidation and subsequent ketogenesis (Fig. 7) and 2) at 6 months of age, after 24 hours of fasting, the cerebellum phospholipid profile was significantly different in HFD-fed individuals (Hq and WT, with various complex phospholipids being more abundant) than CD-fed animals confirming absorption of fats into the central nervous system (CNS) (Fig. 8).

We next analyzed the individual evolution of the rotarod score reported to the initial (1 month) weight. No impact of the initial weight on the rotarod scores in the Hq mice (CD- or HFD-fed animals) could be observed, ruling out the possibility of a weight-associated bias (not shown).

**Figure 9:**
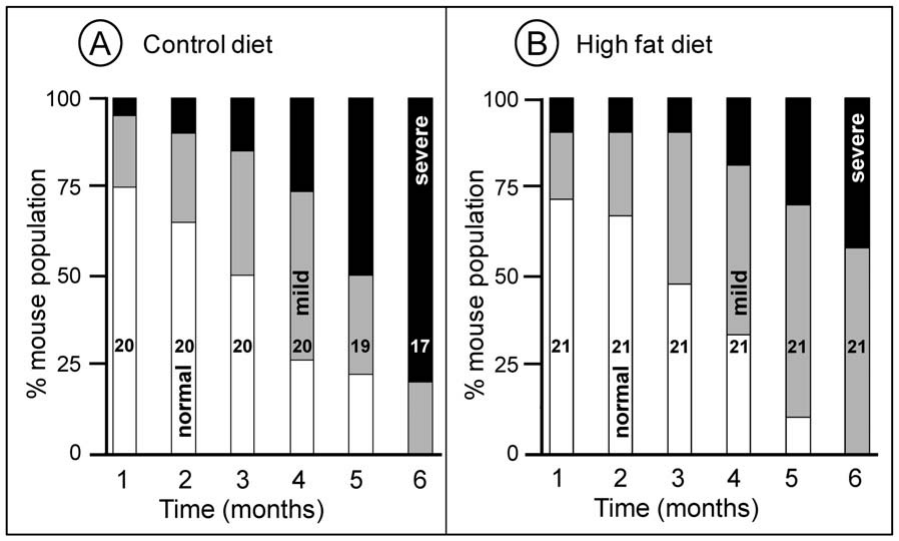
Tail supension test in Harlequin mice fed the control (A) or high fat (B) diet. From 5 months of age, the proportion of less severely affected animals is higher in the HFD group (B). The number (n) of animals is indicated in each bar.

We then analyzed the scores of the tail supension test. In the group of CD-fed Hq animals, the proportion of individuals with a score of 0 (severely impaired) increased from 5% at 1 month of age to 80% at 6 months of age (Fig. 9A). The HFD improved tail suspension test scores in Hq mice suggesting a reduction in the rate of neurodegeneration: only 42% of animals scored 0 after 6 months (Fig. 9B). Similarly, the percentage of individuals with a score of 1 (intermediate severity) was 20% at 1 month of age in both groups and 20% at 6 months of age in the CD-fed Hq animals versus 58% in the HFD-fed Hq group confirming a slower progression of the disease (Fig. 9A and B). The proportion of individuals with a score of 2 (absence of abnormality) was about 75% in both groups at 1 month of age and 0% in both groups at 6 months of age (Fig. 9A and B).

### Cerebellar atrophy and baldness are less severe in HFD-fed Hq mice than in CD-fed Hq animals

**Figure 10:**
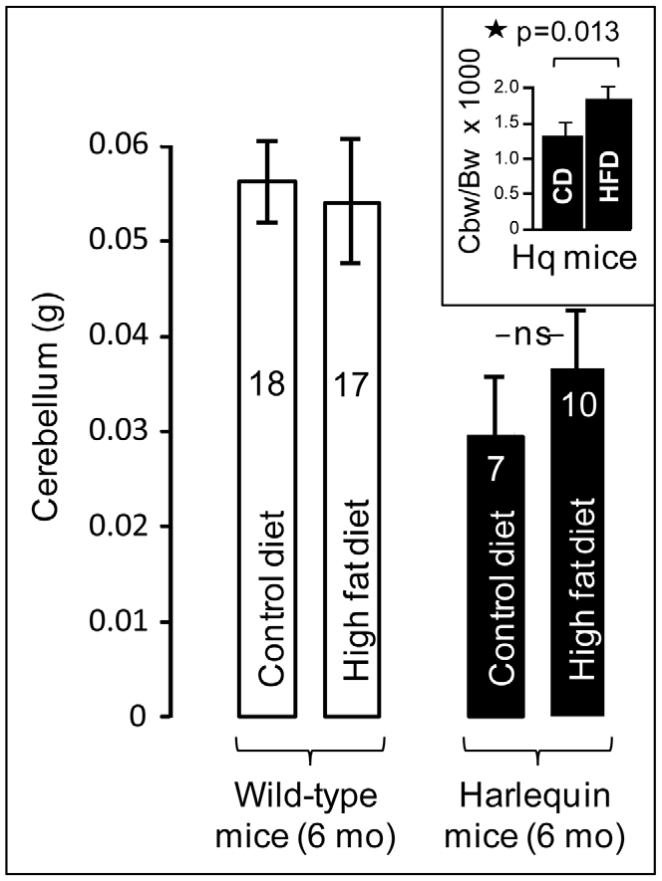
Weight of the dissected cerebella at 6 months of age in wild type (open bars) and Harlequin (dark bars) individuals fed the control or high fat diet. The number of animals is indicated inside each bar. The higher weight of cerebella from HFD-fed Hq animals (compared to CD-fed Hq animals) became significant when ratios of the cerebellar weight (Cbw) to the animal body weight (Bw)×1000 were compared (inset). ns: non-significant.

We next compared the cerebellar weights between the CD- and HFD-fed animal groups. In WT animals, diet had no impact on the cerebellum weight which was significantly higher than in the Hq population (Fig. 10). In comparison, at 6 months of age, the average weights of the cerebella of HFD-fed Hq animals were higher than those of CD-fed Hq mice (Fig. 10). This difference became statistically significant after correction with the body weight (Fig. 10; inset).

Finally, baldness has been shown to be a relevant parameter of disease progression [10]. By scoring baldness in two stages (mild baldness: <66% of body surface or severe baldness: >66% of body surface), we noticed that on average at 6 months of age, the relative proportion of animals with severe baldness was 60% in the CD-fed Hq animals and 38% in the HFD-fed Hq animals.

## Discussion

This study was initiated to determine our ability to evaluate therapeutic options in mice in the context of mt disease with human hallmarks *i.e.* reduced cohorts and strongly variable progression and severity. Because of the well-established ability of fats to induce mitochondrial biogenesis, we decided to test the effect of HFD which is often recommended to RC (especially CI) deficient patients [25], [26], [27], [28]. We first showed that supplementing cell medium with palmitate resulted in protection from cell death caused by glucose withdrawal in three CI-deficient human fibroblasts cell lines. This effect was presumably based on the FA-induced stimulation of mt biogenesis [21], [22], [23], [24], since a number of mt activities were found increased in our CI-deficient cells. We ruled out a potential uncoupling effect of FA on oxidative phosphorylation that could have contributed to protect cells from death both by increasing electron flow in the RC and decreasing superoxide production [34]. Besides, unlike pyruvate [31], palmitate did not improve cell ability to handle hydrogen peroxide insult.

Our study in the Hq mice established that HFD significantly slowed down disease progression regarding major neurodegenerative symptoms (indirectly assessed by rotarod and tail supension tests [35], [36]) and cerebellar atrophy (assessed by cerebellum weight). While this beneficial effect was observed on average in the HFD-fed Hq population, it was even more pronounced in a subset of Hq individuals. This subset population (29%) of responder animals had a preserved or slightly reduced (≤10% of the initial value) score on the rotarod at 6 months while 100% of CD-fed animals had a reduced score at a similar time point. The existence of highly responsive individuals echoes the Hq disease variability [37]. While we still attempt to understand what could confer diet-sensibility to a subset of animals, this suggests that individual therapeutic response is a key-feature of mt disease as reported recently in Leber Hereditary Optic Neuropathy [38].

It is well accepted that in the CNS, fatty acid oxidation (FAO) does not directly participate to energy metabolism [39]. However, CNS-FAO especially in cerebellum [40] could be involved in the metabolism of complex cellular lipids that are vital to neuronal function. This newly described role for FAO in the cerebellum might play a role in the positive effects of HFD in Hq mice.

Recently, the effects of a massively fat-enriched (ketogenic) diet were studied on a mouse model with late-onset progressive mt myopathy [41]; in keeping with our results, these HFD-fed mice exhibited a slower histological disease progression characterized by a decreased amount of cytochrome *c* oxidase negative muscle fibers.

Altogether, our data not only answer our initial questioning on the use of Harlequin mice to investigate therapy, but also pave the way to a number of future researches aiming at 1) qualitatively improving the HFD for instance by modifying its composition; 2) testing more non-invasive tools to better appreciate the efficacy of therapeutic approaches; 3) studying molecular/biochemical/histochemical biomarkers, that, we and others previously found affected during the Hq mouse disease and possibly responsive to HFD.

Finally, we demonstrated that an enriched-fat diet significantly slowed down disease progression in Harlequin mice, thus providing additional solid bases for further clinical evaluation of the HFD in OXPHOS-deficient patients. In contrast to current dogma, moderate numbers of patients with identical disease (20 per group) should be sufficient to obtain statistically robust therapeutic indices in the patient population as far as the potential existence of responders and non responders is recognized. These findings provide a defined reference which will facilitate preclinical and early clinical studies targeting mitochondrial disease.

## Materials and Methods

### Cell culture and cell survival

CI-deficient human fibroblasts previously shown to harbor mutations in the *NDUFS1* gene (2 patients, P1 and P2) [29] and the *NDUFS4* gene (1 patient, P3) [30] were analyzed. Fibroblasts taken with written informed consent from healthy controls and patients were grown under standard conditions in Dulbecco modified Eagle's medium (DMEM; Gibco Invitrogen, Villebon sur Yvette, France) supplemented with 4.5 g/L glucose, 10% of fetal bovine serum, 100 µg/mL penicillin/streptomycin and 4 mM glutamine in T75 flasks until 100% of confluence and then transferred to 6-well plates at an initial density of 15,000 cells/cm^2^. 24 hours later, after counting, wells were washed with phosphate-buffered saline (PBS) and cells cultured in 3 different media: 1) DMEM, 4 mM glutamine; 2) DMEM, 4 mM glutamine, 100 µM palmitate (Sigma-Aldrich, St Quentin Fallavier, France) and 3) DMEM, 4 mM glutamine, 2 mM pyruvate (Sigma-Aldrich, St Quentin Fallavier, France).

In order to allow palmitate entry through plasma cell membrane, it had to be solubilized with fatty-acid free bovine serum albumin (BSA, Sigma-Aldrich, St Quentin Fallavier, France). Stock solutions of palmitate 500 µM/BSA 2.5 mg/mL were prepared as described elsewhere [42]. Palmitate was extemporaneously mixed to culture medium with 1 mM L-carnitine (Sigma-Aldrich, St Quentin Fallavier, France) and 20 mM Hepes (Gibco Invitrogen, Villebon sur Yvette, France) to obtain a 100 µM final palmitate solution.

Cell survival was assessed every 24 hours as follows. Cells were trypsinized and collected. The cells were then incubated with an equal volume of trypan blue and counted using an automated cell counter (Countess, Invitrogen, Villebon sur Yvette, France). The experiments were performed in triplicates.

### Evaluation of cell survival after H_2_O_2_ incubation

Experiments were performed in triplicates in control fibroblasts which were seeded in 6-well plates at 10,000 cells/cm^2^ in 9 different conditions: 1) DMEM, 4.5 g/L glucose, 4 mM glutamine; 2) DMEM, 4.5 g/L glucose, 4 mM glutamine, 100 µM palmitate; 3) DMEM, 4.5 g/L glucose, 4 mM glutamine, 2 mM pyruvate; 4) DMEM, 4.5 g/L glucose, 4 mM glutamine, 0.001 mg/mL glucose oxidase (Sigma-Aldrich, St Quentin Fallavier, France); 5) DMEM, 4.5 g/L glucose, 4 mM glutamine, 0.001 mg/mL glucose oxidase, 100 µM palmitate; 6) DMEM, 4.5 g/L glucose, 4 mM glutamine, 0.001 mg/mL glucose oxidase, 2 mM pyruvate; 7) DMEM, 4.5 g/L glucose, 4 mM glutamine, 0.001 mg/mL glucose oxidase, 350 ng/mL catalase (Sigma-Aldrich, St Quentin Fallavier, France); 8) DMEM, 4.5 g/L glucose, 4 mM glutamine, 0.001 mg/mL glucose oxidase, 350 ng/mL catalase, 100 µM palmitate; 9) DMEM, 4.5 g/L glucose, 4 mM glutamine, 0.001 mg/mL glucose oxidase, 350 ng/mL catalase, 2 mM pyruvate. Cells were counted 24 hours later.

### Enzyme assays

Fibroblasts were cultured under 2 conditions (DMEM 4 mM glutamine; DMEM 4 mM glutamine, 100 µM palmitate) during 48 hours. Then, the cells were trypsinized and centrifuged 5 min×1500 g. The supernatant was discarded and the pellet washed (5 min×1500 g) with 1 mL PBS. The majority of the fresh pellet was used for polarographic assay [43]. A small aliquot of the pellet was deep-frozen in 20–40 µL PBS solution and subsequently thawed using 1 mL of ice-cold solution consisting of 0.25 M sucrose, 20 mM Tris (pH 7.2), 2 mM EGTA, 40 mM KCl and 1 mg/mL BSA, 0.004% digitonin (w/v), and 10% Percoll (v/v) (medium A). After 7 min incubation at ice temperature, cells were centrifuged (5 min×2300 g), the supernatant discarded, and the pellet washed (5 min×2300 g) with 1 mL of medium A devoid of digitonin and Percoll. The final pellet was re-suspended in 20–30 µL of this medium and used for spectrophotometrical enzyme assays.

Respiratory chain enzyme activities were spectrophotometrically measured using a Cary 50 UV–visible spectrophotometer (Varian Inc, Les Ulis, France) [43], [44].

Intact cell respiration and mt substrate oxidation (using 0.004% digitonin-permeabilized cells) were polarographically estimated in a magnetically-stirred 250 µL-cell thermostated at 37°C (DW1 Clark oxygen electrode; Hansatech Instruments; Norfolk, England). All chemicals were of the purest grade available from Sigma-Aldrich (St Quentin Fallavier, France). Protein concentration was measured according to Bradford.

### Animals

Hemizygous (Hq/Y) males were obtained by mating Hq/X females with wild type (WT) males obtained from the Jackson Laboratory (Bar Harbor, ME). All mice used in this study were F1 mice bred from founders having a mixed genetic background (B6CBACaAw-J/A-Pdc8Hq/J). The mice were housed with a 12-h light/dark cycle with free access to food and water.

From weaning (1 month of age), each animal was alternatively assigned to one of the 4 groups: Hq fed the control diet (Hq-CD, n = 20), Hq fed the HFD diet (Hq-HFD, n = 21), WT fed the control diet (WT-CD, n = 21) and WT fed the HFD (WT-HFD, n = 21).

### Ethics Statement

The ethics committee of Robert Debré University Hospital (APHP, 75019 Paris, France) approved the study on human fibroblasts. Informed written consent was obtained from healthy controls, probands (if possible) and parents.

Details of the mouse study were submitted to and approved by the Debré-Bichat Ethics Committee on Animal Experimentation (http://www.bichat.inserm.fr/comite_ethique.htm) - Protocol Number 2010-13/676-0003 - (in accordance with the French law on animal protection).

### Genotype determination

Mice were genotyped using multiplex PCR as already reported [10].

### Phenotypic evaluation

Growth retardation, baldness and cerebellar ataxia have already been studied as relevant markers of disease progression [10] and were monthly studied between one and six months of age. Cerebellar ataxia was assessed using two indirect tests. 1) The rotarod test specifically evaluates coordination and balance [32]. Mice walked on a computer-driven rotating rod (Imetronic; Pessac, France) at speeds that increased from 4 to 40 rpm at 1, 2, 3, 4, 5, and 6 months of age, for 4 consecutive days after training the day before. The time of falling from the rod was recorded. The 3 better of 4 consecutive attempts were kept and the arithmetic mean of these 3 values set aside for further analysis. 2) The tail supension test is a marker of disease progression in a number of mouse models of neurodegeneration [35], including the large group of cerebellar ataxias [36]. Mice were scored as followed: After grasping the tail near its base and lifting the mouse clear of all surrounding objects, the hindlimb position was observed for 10 seconds. If the hindlimbs were consistently splayed outward, away from the abdomen, it was assigned a score of 2, as in WT mouse. If one or both hindlimbs were retracted toward the abdomen for more than 50% of the time suspended, it received a score of 1. If its hindlimbs were entirely retracted and touching the abdomen for more than 50% of the time suspended, it received a score of 0 (the most severe phenotype).

### Diet protocol

HFD was a fat-enriched modification of the CD (R04, SAFE diets, Augis, France). HFD consisted of fat 30 kcal%, carbohydrate 52 kcal% and protein 18 kcal% and CD of fat 8 kcal%, carbohydrate 72 kcal% and protein 20 kcal%. Both diets were designed to be isocaloric with approximately 3000 kcal/kg of chow. CD contained 77% of unsaturated fatty acids (of which 75% were polyunsaturated fatty acids, PUFA) and 23% of saturated fatty acids. HFD contained 54% of saturated fatty acids and 46% of unsaturated (of which 33% were PUFA). Daily chow consumption was measured for 3 consecutive days in four 3-month aged mice of each group.

### β-hydroxybutyrate determination and phospholipid profiling of cerebellum membranes

At 6 months of age, after 24 hours of fasting, the mice were sacrificed by cervical dislocation and cerebellum was dissected and weighted. Blood β-hydroxybutyrate was determined with Optium Xceed (MediSense, ABOTT, Rungis, France).

Phospholipid profiling of cerebellum membranes was performed in 14 Hq individuals (7 HFD-fed and 7 CD-fed) and in 14 WT individuals (7 HFD-fed and 7 CD-fed) as already reported [45]. Cerebella were ground in 1600 µL of 0.1 N HCl and then extracted with 1600 µL of methanol containing internal standards (1.5 mM 1,2-dinonadecanoyl-sn-glycero-3-phosphocholine; 0.25 mM 1,2-sn-diphytanoyl-glycero-3-phosphoethanolamine; 0.25 mM N-heptadecanoyl-D-erythro-sphingosylphosphorylcholine) and 1600 µL of chloroform. After intense stirring for 10 minutes and 1 hour of settling, the solution was centrifuged at 6000 g for 5 minutes. The lower organic phase was extracted and evaporated to dryness under nitrogen gas stream. The dry residue was taken up in 100 µL of chloroform and diluted with 900 µL methanol. 100 µL of this solution were then injected into a liquid chromatography coupled to tandem mass spectrometry (LC/MS-MS) analyzer to quantify various phospholipids classes (PC, phosphatidylcholine; PE, phsophatidylethanolamine; PI, phosphatidylinositol; PS, phosphatidylserine; SM, sphingomyeline).

### Statistics

We used a two-factor, repeated-measures ANOVA (STATVIEW 5.0, SAS Institute, Cary, NC) to test for significant main effects and interactions between main effects. Where ANOVA was significant, t tests were applied a priori. P≤0.05 was considered significant. Values are means ±SEM.
